# Factorial Design as a Tool for the Optimization of PLGA Nanoparticles for the Co-Delivery of Temozolomide and O6-Benzylguanine

**DOI:** 10.3390/pharmaceutics11080401

**Published:** 2019-08-10

**Authors:** Maria João Ramalho, Joana A. Loureiro, Manuel A. N. Coelho, Maria Carmo Pereira

**Affiliations:** LEPABE—Laboratory for Process Engineering, Environment, Biotechnology and Energy, Faculty of Engineering, University of Porto, Rua Dr. Roberto Frias, 4200-465 Porto, Portugal

**Keywords:** drug delivery, experimental design, fractional factorial design, O6-methylguanine DNA methyltransferase (MGMT) protein, glioblastoma multiforme

## Abstract

Poly(d,l-lactic-*co*-glycolic) (PLGA) nanoparticles (NPs) have been widely studied for several applications due to their advantageous properties, such as biocompatibility and biodegradability. Therefore, these nanocarriers could be a suitable approach for glioblastoma multiforme (GBM) therapy. The treatment of this type of tumours remains a challenge due to intrinsic resistance mechanisms. Thus, new approaches must be envisaged to target GBM tumour cells potentially providing an efficient treatment. Co-delivery of temozolomide (TMZ) and O6-benzylguanine (O6BG), an inhibitor of DNA repair, could provide good therapeutic outcomes. In this work, a fractional factorial design (FFD) was employed to produce an optimal PLGA-based nanoformulation for the co-loading of both molecules, using a reduced number of observations. The developed NPs exhibited optimal physicochemical properties for brain delivery (dimensions below 200 nm and negative zeta potential), high encapsulation efficiencies (EE) for both drugs, and showed a sustained drug release for several days. Therefore, the use of an FFD allowed for the development of a nanoformulation with optimal properties for the co-delivery of TMZ and O6BG to the brain.

## 1. Introduction

Nanomedicine has been arousing increasing interest, since it allows the early diagnosis and monitoring of several diseases and also can increase the efficacy of conventional pharmacological treatments by enabling their controlled delivery [1,2,3,4]. Several materials have been widely studied for nanoparticles (NPs) development. Among polymeric materials, poly(d,l-lactic-*co*-glycolic) (PLGA) is perhaps the most used, due to being FDA-approved, biocompatible, biodegradable, and having tunable physicochemical properties [5]. Since these polymeric NPs allow the release of drugs in a controlled and sustained manner for long periods, the required drug doses and administration frequency can be minimized, decreasing the toxicity of the encapsulated drug [6]. Additionally, PLGA NPs are up-taken by endocytic mechanism, circumventing the p-glycoprotein-mediated cellular efflux, enabling drug accumulation in the target cells. Also, since this polymer can be easily functionalized with different materials, the design of NPs with diverse targeting moieties can be achieved [7]. 

Therefore, several efforts have been conducted in seeking the development of PLGA NPs for novel therapies with several therapeutic drugs without reducing their bioavailability or activity. The entrapment of therapeutic drugs in PLGA NPs also allow a reduction in their toxicity, and therefore these NPs have been extensively studied for cancer treatment. Most particularly, several attempts to encapsulate temozolomide (TMZ) in PLGA NPs for brain delivery have been conducted [8]. TMZ, the gold standard treatment for glioblastoma multiforme (GBM), presents several limitations, as it has low bioavailability and high toxicity, reducing its pharmacological activity [9]. Therefore, TMZ encapsulation in a nanocarrier emerges as a suitable approach to increase its chemotherapeutic efficacy, since it will avoid drug elimination after administration and increase its accumulation in the target tissues. GBM is the brain cancer with a high mortality rate, since its classical therapy fails to effectively cure the disease [10]. Thus, it is urgent to find new approaches for its treatment. 

In fact, different PLGA delivery nanosystems were developed for TMZ in the last decade. Jain et al., 2014 developed PLGA NPs for TMZ entrapment. Although exhibiting promising results, modifying the NPs’ surface with a targeting moiety to improve the specificity of the nanocarriers could be a suitable strategy to enhance its efficiency [11]. Later, Ananta et al., 2016 prepared PLGA NPs that were not able to maintain a favorable sustained release of TMZ [12]. Also, some authors used active targeting strategies to increase the NPs specificity to GBM cells. In fact, Jain et al., 2011 modified the surface of PLGA NPs with transferrin molecules to promote transport across the blood–brain barrier (BBB). The developed nanocarriers enhanced the accumulation and cytotoxic effect of TMZ in mice’s brains [13]. Lee et al., 2016 also functionalized PLGA NPs with folate molecules to increase the NPs accumulation in target cells, but the prepared nanosystems did not exhibit good encapsulation efficiency (EE) values [14]. Our group (2018) also developed anti-transferrin receptor monoclonal antibody modified PLGA NPs for the delivery of TMZ. The obtained results showed that PLGA NPs are a promising approach for GBM treatment with TMZ [15]. 

However, GBM patients usually exhibit low sensitivity to therapy due to intrinsic resistance mechanisms. The O6-methylguanine DNA methyltransferase (MGMT) protein is highlighted as one of the main causes of therapeutic failure of TMZ [16]. Therefore, new strategies to decrease resistance to therapy are necessary. A promising approach is the use of molecules that are able to revert or inhibit these intrinsic resistance mechanisms. Most recently, co-treatment with O6-benzylguanine (O6BG) has been explored to decrease the resistance to TMZ’s therapy by binding to the MGMT protein, leading to its inhibition, and consequently hindering the repair of the damaged DNA [17,18]. Nanoencapsulation of O6BG may reduce its toxicity in healthy tissues by targeted delivery. Accumulation of O6BG in healthy tissues is undesirable to avoid inactivation of the MGMT protein in these tissues, and consequently exacerbates the toxicity of alkylating agents, such as TMZ [19]. 

Also, systemic administration of two free drugs usually leads to infective pharmacological activity and, consequently, treatment failure, due to differences in the biodistribution profile of each drug [20]. Therefore, the entrapment of both drugs in NPs should address this problem. In fact, to increase therapeutic efficiency, the co-loading of TMZ with other molecules in PLGA NPs has also already been studied for glioblastoma therapy. Xu et al., 2016 proposed the co-delivery of TMZ with paclitaxel using PLGA NPs [21]. Until this moment, to the best of our knowledge, the co-encapsulation of TMZ and O6BG in a nanosystem was not reported. Thus, the aim of this work was to prepare a nanocarrier for the co-loading of both drugs.

However, the use of PLGA NPs faces a few limitations because of their poor loading capacity and the typical initial burst release. Additionally, the production of PLGA NPs requires different stages that can present high costs and be difficult to scale-up, such as centrifugation and dialysis. Also, it can be challenging to entrap hydrophilic drugs, since those exhibit a high partition into the aqueous phase during NPs preparation [6]. Therefore, is necessary to optimize the preparation methods. As well, usually the entrapment of two distinct molecules, with different physicochemical properties such as hydrophilicity and molecular weight, can be a challenging task. Though, high encapsulation of both drugs is desirable to increase the nanosystem efficiency and to reduce the amount of administered polymer. Also, it is essential to control the physicochemical properties of the developed NPs, such as size and surface charge, since these parameters control their biological fate, biodistribution, and toxicity, therefore affecting their therapeutic potential [22,23].

Therefore, experimental design could be a suitable approach to optimize the nanoformulation. In fact, in the last years, experimental design has been used for the optimization of drug-loaded NPs [24,25]. This is because a nanoformulation design requires full knowledge of the correlation between the experimental factors and the obtained NPs properties. To obtain an optimized nanoformulation using a conventional screening method (evaluating the effect of one experimental variable at a time) is expensive and time-consuming. Experimental design is therefore a validated and useful tool for the development and optimization of experimental procedures with a lower number of observations while still providing the desired information on the correlation between the experimental and the response variables. The obtained model can then be used for predicting future observations within the original design range [26]. Different types of experimental designs can be used, and in this work, a fractional factorial design (FFD) is proposed. When several experimental variables are being studied, FFD is a rapid and reliable tool, allowing the exploration of a maximum number of variables, while requiring less experimental observations, and still obtaining the desired information. However, since it uses only a partial combination of factors, some information about possible interactions can be lost [27].

Thus, the effect of five experimental variables on the physicochemical properties of the developed PLGA NPs and encapsulation efficiency (EE) of both drugs was studied in this work. The studied experimental factors were the amounts of TMZ, O6BG, surfactant, organic solvent, and polymer. 

## 2. Materials and Methods 

### 2.1. Materials

TMZ (MW 194.15, purity ≥99%) was purchased from Selleck Chemicals (Munich, Germany). O6BG (MW 241.25, purity ≥98%) was obtained from Abcam (Cambridge, UK). PLGA Resomer^®^ RG503H (50:50; MW 24,000–38,000), poly(vinyl alcohol) (PVA) Mowiol^®^ 4-88 (MW 31,000), phosphate buffer saline (PBS) and dichloromethane were acquired from Sigma–Aldrich (St. Louis, MO, USA). Uranyl acetate was provided by electron microscopy sciences (Hatfield, UK). 

### 2.2. Preparation of TMZ+O6BG-Loaded PLGA NPs

For the synthesis of PLGA NPs loading, both TMZ and O6BG, a variation of the single emulsion–solvent evaporation method, were used [28]. Known amounts of PLGA, TMZ, and O6BG were dissolved in a dichloromethane solution. A 2% (*w*/*v*) PVA solution was added drop-by-drop to the prepared organic solution. Then, the solution was vortexed (Genius 3, ika^®^vortex, Germany) and placed in an ultrasonic bath at an ultrasonic frequency of 45 kHz (Ultrasonic cleaner, VWR^TM^, Kuala Lumpur, Malaysia) to yield an oil-in-water (o-in-w) emulsion.

The emulsion was then poured into a 0.2% (*w*/*v*) PVA solution and maintained in unremitting agitation in a magnetic stirrer (800 rpm, Colorsquid, ika^®^, Staufen, Germany) until complete organic solvent evaporation (6 h). The suspension was then filtered using a membrane with a pore size of 200 nm, (polyethersulfone membrane syringe filter, VWR, Radnor, PA, USA) and stored at 4 °C overnight to increase the NPs’ stability, avoiding their aggregation. After, the samples were centrifuged for 30 min at 14100× *g* (MiniSpin^®^plus, Eppendorf, Hamburg, Germany), to separate NPs from the non-encapsulated drug. The supernatant containing the non-encapsulated drug was saved for analysis.

### 2.3. Experimental Design and Data Analysis 

High and comparable EE values for each drug are a prerequisite for the co-loading in the same NPs. In preliminary studies, it was verified that the co-encapsulation of TMZ and O6BG reduced the encapsulation of each drug alone. Thus, it was necessary to optimize the entrapment of both drugs. Also, it was important to control the physicochemical properties of the developed NPs, since these parameters influence the therapeutic efficacy and toxicity of the NPs. To obtain an optimized formulation, it is useful to study how the several experimental parameters influence the entire production process.

Therefore, a 2^5-2^ FFD was implemented using the Minitab Statistical Software (Minitab Inc., State college, PA, USA) to determine the effect of different experimental factors on the PLGA NPs features. The studied independent variables were the amount of both used drugs, the quantity of surfactant and organic solvent, and the amount of polymer. A variation of a full factorial design, in which only a subset of the total runs was performed, took place. The chosen factors of interest were varied on two levels (determined in preliminary studies) according to the experimental plan presented in Table 1. Two replicates were conducted for each combination and for center levels, and the order of the experiments was randomly sorted to avoid any bias. 

The observed response dependent variables were the NPs size, PdI, zeta potential values, and EE of both TMZ and O6BG. For the experimental design, 18 formulations were prepared, and an outline of the experimental plan and its results is shown in Table 2.

The applied experimental design accounts for main terms and two-factor interactions terms. The latter refers to two different variables that interact with each other, creating a combined effect on the response that independently would not occur. Therefore, the main effects and the two-factor interactions are accounted for in the used regression model. Thus, regression equations were obtained for each studied dependent variable to quantify the relationship between these and all the experimental independent variables. The experimental data was then fitted to the following polynomial regression Equation (1) [29]:

(1) $$Y=\beta_{0}+\;\overset{5}{\underset{i=1}{\sum }}\beta_{i}X_{i}+\;\sum \beta_{ij}X_{i}X_{j}$$

in which *Y* is the predicted response; *β_0_* is the intercept term and the remaining term; *X_i,j_* is the studied levels of the independent variables; and *β*_j,i_ is the fitted coefficients for *X_i,j_*.

The statistical regression models for the different dependent variables were fitted independently. Additionally, the polynomial equations were statistically validated using ANOVA (Analysis of Variance) by statistical significance of coefficients, *R^2^* values, and normal distribution of the residues. Minitab Statistical Software was also used for the statistical analysis of the data. 

### 2.4. TMZ+O6BG-Loaded PLGA NPs Physicochemical Characterization 

#### 2.4.1. Dynamic Light Scattering for Size Determination

The mean diameter and size distribution of the prepared NPs were evaluated by dynamic light scattering (DLS). The measurements were performed in a ZetaSizer Nano ZS (Malvern Instruments, Worcestershire, UK). The attained data is given in intensity distribution. The intensity-weighted mean diameter (Z-average) is given. For the optimized formulation, at least three independent measurements were performed, and obtained results are expressed as the mean and standard deviation (SD). Statistical analysis was performed using the *t*-student test, and *p*-values ≤ 0.05 were considered significant.

#### 2.4.2. Laser Doppler Velocimetry Method for Zeta Potential Determination

The zeta potential values of the prepared NPs were determined by laser doppler velocimetry method. The measurements were also performed in a ZetaSizer Nano ZS (Malvern Instruments, Worcestershire, UK). The analysis was performed using the dielectric constant of water. For the optimized formulation, at least three independent measurements were performed, and obtained results are expressed as the mean and SD. Statistical analysis was performed using the *t*-student test.

#### 2.4.3. Transmission Electron Microscopy for Morphological Analysis 

The morphological analysis of the NPs was obtained by transmission electron microscopy (TEM). The NPs were prepared on copper grids (Formvar/Carbon-400 mesh Copper, Agar Scientific, Essex, UK) and negatively stained. For that, 10 μL of samples were stained with 2% (*v*/*v*) uranyl acetate for 45 s, and air-dried. This is a heavy metal salt able to scatter electrons, enhancing the contrast to better visualize the samples [30]. Then, the NPs were visualized using a JEM 1400 electron microscope (Jeol, Tokyo, Japan) at an accelerating voltage of 80 kV.

### 2.5. TMZ+O6BG-Loaded PLGA NPs Stability Studies

The stability of the prepared PLGA NPs was analysed through size and zeta potential variations. PLGA NPs’ dispersions in ultrapure water were stored at 4 °C and DLS measurements were performed at different timepoints to evaluate variations in PLGA NPs size and zeta potential values. These measurements were performed weekly, for 6 weeks. For optimized nanoformulation, three independent samples were used.

### 2.6. Drug Encapsulation Efficiency of TMZ+O6BG-PLGA NPs

The TMZ and O6BG EE values of the prepared PLGA NPs were assessed by UV–Vis spectrophotometry, using the following Equation (2):

(2) $$EE=\;\frac{total\;amount\;of\;drug-\;amount\;of\;free\;drug}{total\;amount\;of\;drug}\;\times \;100$$

Non-encapsulated TMZ and O6BG molecules were separated from the NPs colloidal suspension by centrifugation (30 min, 14100× *g*, MiniSpin^®^plus, Eppendorf, Germany) and quantified (UV-1700 PharmaSpec UV-Vis spectrophotometer, Shimadzu, Kyoto, Japan) at 240 nm and 329 nm for O6BG and TMZ, respectively. The results were correlated to control samples corresponding to total amount of drug. For the optimized formulation, three independent experiments were conducted, and obtained results are expressed as the mean and SD. Statistical analysis was performed using the *t*-student test.

### 2.7. In Vitro Release of TMZ and O6BG from PLGA NPs 

To assess the drug release profile of the developed NPs, in vitro dialysis studies were performed for 20 days at 37 °C. For that, a cellulose dialysis membrane (Float-A-Lyzer G2, CE, 10KDa, SpectrumLabs, Los Angles, CA, USA) was rinsed in ultrapure water for 24 h before the beginning of the experiments and equilibrated with release medium 1 h before the dialysis. 

A known amount of TMZ+O6BG-PLGA NPs diluted in 2 mL of release medium was placed into the inner space of the dialysis membrane. The outside space was filled with a known volume of release medium to ensure sink conditions. PBS (pH 7.4, 0.01 M) was used as the release buffer to mimic the physiological salt concentrations and pH of blood plasma. The dialysis membrane was kept in continuous stirring at 200 rpm at 37 °C, simulating the physiological temperature. The amount of drug release at predetermined timepoints (0, 6, and 24 h; and day 3, 6, 8, 9, 11, 13, 16, and 20) was quantified by UV-Vis spectrophotometry (UV-1700 PharmaSpec UV-Vis spectrophotometer, Shimadzu, Kyoto, Japan). A solution of TMZ and O6BG in PBS was used as control. For the optimized formulation, three independent experiments were conducted, and obtained results are expressed as the mean and SD. Statistical analysis was performed using the *t*-student test.

The TMZ and O6BG release curves, representing the percentage of drug released in function of time, were then plotted by the following Equation (3):

(3) $$\%\;drug\;released=\frac{amount\;of\;drug\;released\;at\;time\;t}{amount\;of\;encapsulated\;drug}\times \;100$$

## 3. Results and Discussion

### 3.1. Statistical Analysis of Experimental Data

The applied experimental design allowed the identification of the experimental factors influencing the physicochemical properties of the NPs, astheir size and the encapsulation efficiency of both drugs on the polymeric matrix. Since zeta potential (≤−20 mV) and PdI values (<0.2) were always inside the desired range, they were not considered for this model.

Previous nanoformulation studies were conducted to identify the five major experimental variables that affect the PLGA NPs properties, such as size and EE of both drugs. The amount of TMZ, O6BG, surfactant, organic solvent, and polymer were chosen and varied in two levels. The levels choice was based on the knowledge acquired in the preliminary experiments. All other parameters, such as type of surfactant and organic solvent, aqueous to organic phase ratio, time of sonication, ultrasonic frequency of sonication, process temperature, and emulsification and evaporation processes, were maintained constantly.

The mathematical model (Equation (1)) was fitted to the data and statistical analysis was performed using ANOVA (Table 3). As Table 3 shows, the model is statistically significant (*p* < 0.05) with insignificant lack of fit (*p* > 0.05) for all the chosen responses. Hence, the linear model was acceptable for response prediction within the range of experimental variables. 

**Figure 1:**
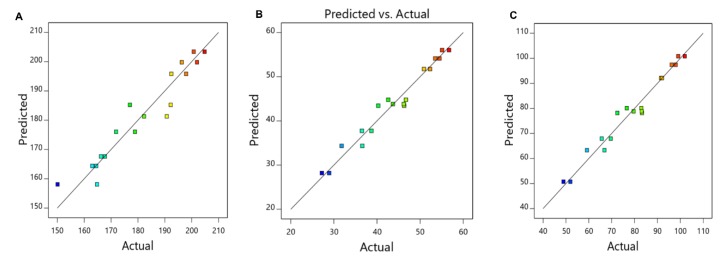
Graphical plots of the experimental values versus the model predicted values for (**A**) mean size, (**B**) EE for TMZ, and (**C**) EE for O6BG. Red dots indicate high values and blue dots low values, respectively.

Figure 1 also shows a satisfactory agreement between experimental observations and predicted response, proving that the applied regression model is suitable for the determination of the optimal experimental settings for the preparation of NPs.

The statistical analysis of the obtained results also allowed the determination of the regression coefficient (RC) values that describes the direction of the relationship between an experimental variable and the variable response. A negative sign before a factor indicates that the response decreases, whereas a positive sign shows that the response increases with the experimental factor. With the RC values, it was possible to obtain the polynomial regression equation for each studied response, quantifying the relationship between each of the studied experimental variables and the response. The non-significant factors (*p* > 0.05) were excluded from the mathematical model, allowing the reduction in complexity of the attained equations.

Response surface analysis and contour graphs were plotted based on the determined model polynomial function in a three and a two-dimensional model, respectively, illustrating the effect of the chosen significant independent factors on each observed response. The effect of all the independent variables on each dependent variable was studied and the effects of the most significant variables are discussed in detail below.

#### 3.1.1. Effect on NPs’ Size

The size of the NPs is a critical property, influencing its half-life, biodistribution, and cellular internalization. The size of the NPs ranged from 162 nm (sample 7) to 205 nm (sample 12). Almost all the studied variables significantly affected the size of the NPs (*p* < 0.05), as shown in Table 3.

The polynomial Equation (4) describes the relationship between the significant studied experimental variables (*p* < 0.05) and the size of the NPs:

(4) $$\begin{array}{ll} Size= & 134.9-9.67\;amount\;TMZ\;\left(mg\right)+\;83.9\;amount\;O6BG\;(mg)\\ & +\;15.05\;amount\;PVA\;\left(\%\right)+\;1.644\;amount\;PLGA\;\left(mg\right)\\ & -13.41\;amount\;O6BG\;\left(mg\right)\;\times \;amount\;PVA\;\left(\%\right)\\ & -\;2.541\;amount\;O6BG\;\left(mg\right)\;\times \;amount\;PLGA\;\left(mg\right) \end{array}$$

The experimental factor that most significantly affected NPs size was the amount of surfactant, which exhibited a positive effect on the size of the NPs. Thus, an increase in PVA concentration led to an increase in NP size. Although using a high quantity of surfactant can induce the creation and stabilization of smaller NPs due to a reduction of the interfacial tension between the polymer and the external aqueous phase [31], the opposite effect was verified. This may be explained by the increased viscosity of the aqueous phase when increasing the PVA concentration. A higher viscosity decreases the shear stress, originating emulsion droplets with larger sizes. Also, higher amounts of surfactant can promote the coalescence of the NPs, yielding NPs with larger diameters [32]. Also, some studies report that residual PVA remains at the surface, contributing to the size increase [33]. 

Additionally, the amount of PLGA also exhibited a significantly positive effect on the size of the NPs (*p* < 0.05). Increasing the polymer amount increases the viscosity of the organic phase, decreasing the shear stress, as mentioned above. Also, the augmented viscosity hampers the diffusion of the organic solvent into the aqueous phase, leading to the formation of larger emulsion droplets, originating larger NPs after solvent evaporation [34].

Higher initial loading of O6BG positively affected the NPs size (*p* < 0.05), since it will result in higher drug loading, as will be discussed later. The contrary was observed for TMZ amount (*p* < 0.05), since higher amounts of TMZ will decrease its entrapment, as will also be discussed later.

**Figure 2:**
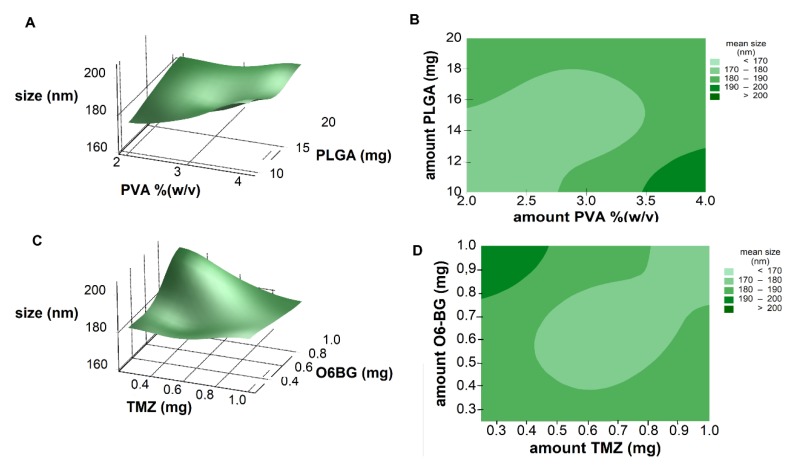
(**A**) Response surface plot and (**B**) contour plot showing the effect of two factors (amount of PVA and PLGA) on the resulting NPs size. (**C**) Response surface plot and (**D**) contour plot showing the effect of two factors (amount of TMZ and O6BG) on the resulting NPs size.

Response surface analysis and contour graphs were plotted (Figure 2) based on this model polynomial function. Contour and surface response plots allowed visual identification of the optimal levels of each factor, to choose the most suitable values for the development of an optimal formulation. Both response surface and contour plots (Figure 2A,B) showed that the lower the amount of PVA and PLGA, the lower the NPs size, as already predicted by the calculated positive RC values (Equation (4)). Thus, optimal nanoformulation with small dimensions would fall into the low and central levels of both factors.

**Figure 3:**
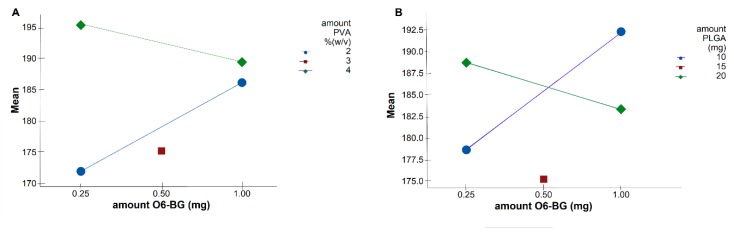
Interaction plots for size data means showing significant two-way interaction terms for the independent variables. Solid blue lines display factor at low level, whereas dashed green lines are the high level of the factors. (**A**) interaction term X_2_X_3_: amount of O6BG/amount of surfactant, (**B**) X_2_X_5_: amount of O6BG/amount of PLGA.

In addition to all the five factors significantly affecting the NPs size, some significant two-factor interactions were observed (Table 3). Interaction plots on Figure 3 show high interaction between two factors. Each point in the interaction plot shows the mean size values at different combinations of factor levels. As the lines are not parallel, with different slopes, the plot indicates that there is an interaction between the two factors [35]. The same is verified in all the attained interaction plots.

The main effects can be confused with the two-factor interactions, which explains the results obtained in Figure 2. Although Equation (4) predicts that the lower the amount of TMZ and the higher the amount of O6BG, the higher the NPs size, optimal values fall into high levels of both TMZ and O6BG. As shown in Figure 3A,B, at low levels of both PLGA and PVA amounts, O6BG positively affects the size of the NPs. On the other hand, at high levels of PLGA and PVA, increasing the O6BG amount decreases the size of the NPs.

#### 3.1.2. Effect on the Encapsulation Efficiency of TMZ

High encapsulation of the drug is desirable to increase the nanosystem efficiency and to reduce the amount of administered polymer. The EE values for TMZ in the prepared NPs ranged from 27.2 (sample 10) to 56.7% (sample 4). 

The polynomial Equation (5) describes the relationship between the significant studied experimental variables (*p* < 0.05) and the EE of TMZ:

(5) $$\begin{array}{ll} EE\%\;TMZ= & 11.84-8.80\;amount\;TMZ\;\left(mg\right)-\;3.40\;amount\;PVA\;\left(\%\right)\\ & +\;7.95\;amount\;DCM\;\left(mL\right)+\;1.461\;amount\;PLGA\;\left(mg\right)\\ & -13.87\;amount\;O6BG\left(mg\right)\;\times \;amount\;PVA\;\left(\%\right)\\ & -\;2.153\;amount\;O6BG\;\left(mg\right)\;\times \;amount\;PLGA\;\left(mg\right) \end{array}$$

EE values were significantly influenced by almost all the studied independent variables, as shown by the calculated *p*-values on Table 3. All the experimental variables show a positive effect on the EE of TMZ, except PVA concentration and TMZ amount. PLGA had a positive effect on the EE of TMZ. As already mentioned, a higher amount of PLGA polymer results in larger NPs and, consequently, higher encapsulation of the drug. Also, the increased viscosity caused by higher PLGA concentration mentioned above could complicate the diffusion of TMZ molecules into the aqueous phase, enhancing the drug’s entrapment into the NPs’ polymeric matrix [36]. On the other hand, increased volumes of organic solvent increased drug solubility, therefore increasing drug entrapment. 

Also, the encapsulation of TMZ decreased with PVA concentration due to a higher partition of TMZ molecules into the aqueous phase during emulsification, decreasing the EE values. It is reported that drug molecules can diffuse out from the oil nanodroplets and solubilize in PVA micelles at the aqueous phase [37]. Increasing the TMZ amount may cause saturation of the organic phase, leading to the partition of the drug molecules to the aqueous phase, lowering its entrapment in the polymeric matrix.

**Figure 4:**
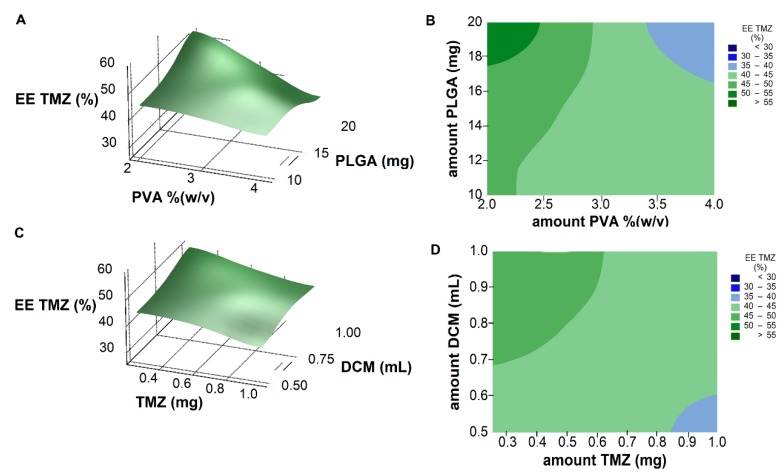
(**A**) Response surface plot and (**B**) contour plot showing the effect of two factors (amount of PVA and PLGA, respectively) on the resulting EE TMZ values. (**C**) Response surface plot and (**D**) contour plot showing the effect of two factors (amount of TMZ and DCM, respectively) on the resulting EE TMZ values.

Response surface analysis and contour graphs (Figure 4) show that the optimal formulation would be prepared with low amounts of PVA and TMZ, and high amounts of PLGA and organic solvent.

**Figure 5:**
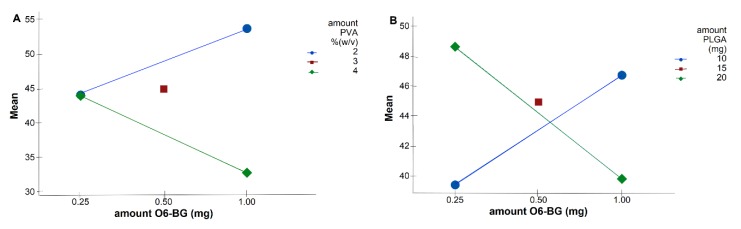
Interaction plots for EE of TMZ data means showing significant two-way interaction terms for the dependent variables. Solid blue lines display factors at a low level, whereas green dashed lines are the high level of the factors. (**A**) interaction term X_2_X_3_: amount of O6BG/amount of surfactant, (**B**) X_2_X_5_: amount of O6BG/amount of PLGA.

All five studied variables were part of an extensive interaction system. A significant two-factor interaction of O6BG amount with both PLGA and PVA amounts (Table 3) was verified (*p* < 0.05), as shown in Figure 5.

The described two-factor interaction between the PVA and O6BG amounts shows a significant PVA concentration effect and interaction term (Figure 5A). There is a difference among the means of the two PVA levels, but not a difference in the means among O6BG amount. This proves that although the amount of O6BG did not show a significant direct effect on the EE values, the interaction term exists. The amount of O6BG affected the nanoformulation at both low and high PVA amounts. At low PVA levels, the O6BG amount showed a positive effect in the TMZ encapsulation. The opposite was observed at high PVA amounts.

The same is verified in the two-factor interaction between the PLGA and O6BG amounts (Figure 5B).

#### 3.1.3. Effect on the Encapsulation Efficiency of O6BG

The EE values of O6BG in the prepared NPs ranged from 70.9 (sample 3) to 99.8% (sample 11). 

The polynomial Equation (6) describes the relationship between the significant studied experimental variables (*p* < 0.05) and the EE of O6BG:

(6) E% O6BG= 104.4− 4.12 amount PVA (%)+ 0.854 amount PLGA (mg)

PVA and PLGA amounts were the only studied independent variables that significantly influenced the EE values, as shown by the calculated *p*-values on Table 3. While PLGA concentration showed a positive effect on the EE of O6BG, PVA concentration exhibited a negative influence. In fact, increasing PVA concentration hampered the encapsulation of the drug, since it enhanced the partition of O6BG molecules into the aqueous phase during emulsification, decreasing the encapsulation efficiencies values [37]. Also, as already mentioned, higher amounts of PLGA polymer resulted in larger NPs and, consequently, higher encapsulation of the drug. Also, the increased viscosity caused by higher PLGA concentration mentioned above could complicate the diffusion of O6BG molecules into the aqueous phase, enhancing the drug’s entrapment into the NPs’ polymeric matrix [36].

**Figure 6:**
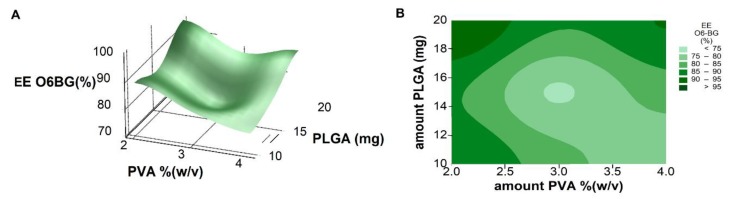
(**A**) Response surface plot and (**B**) contour plot showing the effect of two factors (amount of PLGA and PVA, respectively) on the resulting EE values.

Response surface analysis and contour graphs (Figure 6) show that predicted optimal nanoformulation with high O6BG molecules entrapment would fall into the high levels of PLGA and low levels of surfactant concentration. No significant two-factor interactions were observed.

### 3.2. Nanoformulation Optimization and Physicochemical Characterization 

The optimization process was performed by determining the optimal experimental values, which were obtained by solving the three determined polynomial regression equations and grid searching in the response surface graphs or contour plots applying the following criteria: to minimize the particle size (Y_1_), and to maximize the EE values for both drugs (Y_2_ and Y_3_). The optimum levels of the formulation factors are presented in Table 4. 

A formulation checkpoint was prepared according to the predicted model to validate the reliability and the precision of the factorial design, using these optimal experimental conditions. The checkpoint formulation was prepared in triplicates and the properties of the attained NPs were within the range of the predicted values, as shown in Table 5.

Higher encapsulation verified for O6BG can be explained by its greater affinity for the organic phase, compared with TMZ. While TMZ exhibits a log P value of 0.36, for O6BG, log P is 1.66 (values obtained from Marvin Sketch Calculator software. Chemaxon^TM^,Version 16.4.25, Budapest, Hungary).

**Figure 7:**
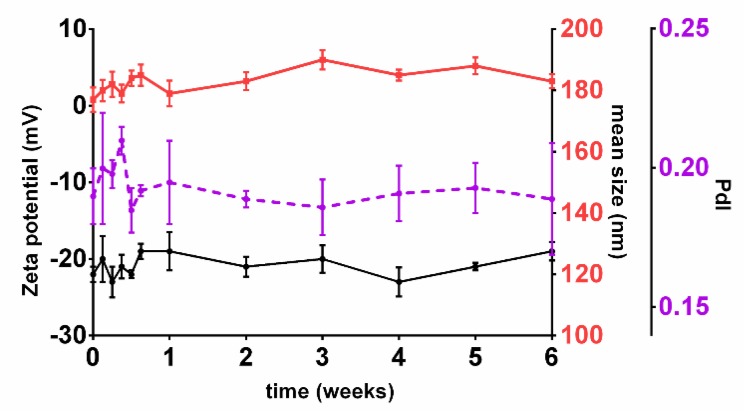
Stability of the prepared checkpoint nanoformulations. Black line: Graphical representation of variations in zeta potential values over 6 weeks (data plotted on the left Y-axis); Red line: Graphical representation of variations in mean sizes over 6 weeks (data plotted on the first right Y-axis); Purple line: Graphical representation of variations in PdI values over 6 weeks (data plotted on the second right Y-axis). Results are given as mean ± SD (*n* = *3*).

The developed NPs exhibit mean dimensions suitable for brain delivery. Although PdI and zeta potential values were not considered for the model, they were also determined for the checkpoint formulations. The developed NPs exhibited zeta potential values of −22 ± 1 mV and PdI values of 0.19 ± 0.01. The high negative zeta potential values are associated with low toxicity [38] and suggest that the NPs are stable [39]. In fact, all the nanoformulations prepared in the 18 runs of the FFD (data not shown) and the checkpoint formulations proved to be stable in storage conditions for at least six weeks, as shown in Figure 7. 

**Figure 8:**
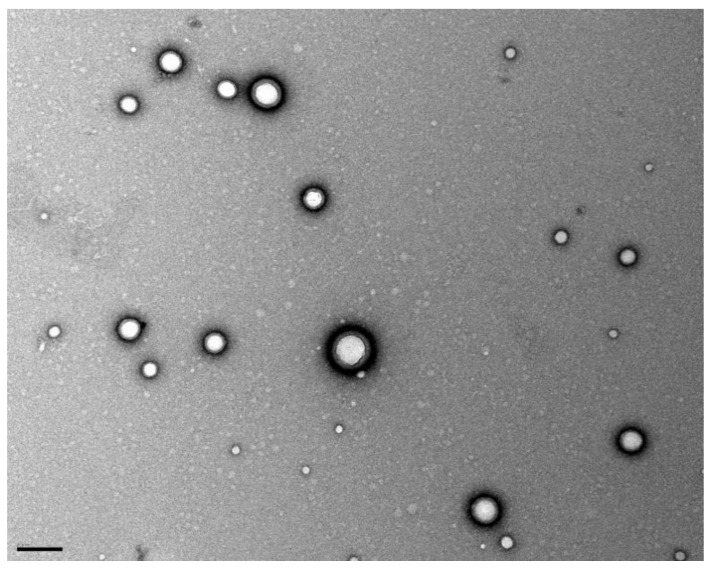
TEM photograph showing the morphology of the developed PLGA NPs. Scale bar is 200 nm.

The stability of the prepared colloidal suspension NPs is granted by a PVA layer on the NP’s surface. During the NPs preparation, the PVA molecules adsorb on the surface of the resultant nanodroplets acting as a mechanic barrier that prevent coalescence and avoid NPs’ aggregation [40]. TEM image (Figure 8) shows this stabilizer layer around the NPs’ surface. Also, the attained PdI values suggest that the colloidal suspension is monodisperse. Therefore, it can be concluded that the method followed for the preparation of PLGA NPs produced well-stabilized monodisperse O6BG+TMZ-loaded PLGA NPs.

### 3.3. Drug Release from the PLGA NPs

**Figure 9:**
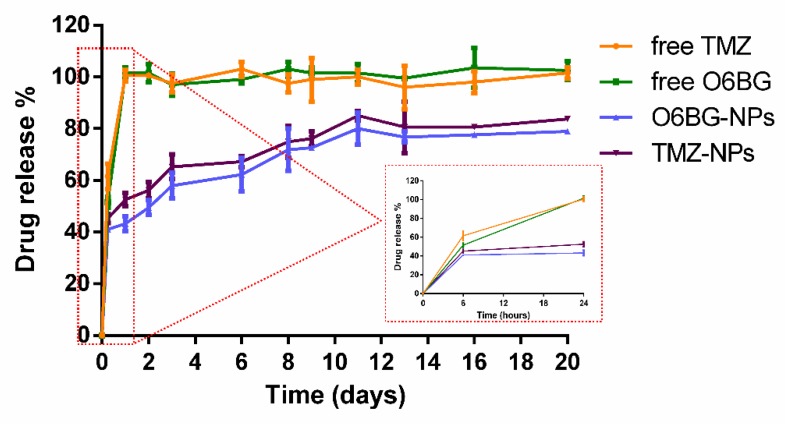
In vitro release of TMZ and O6BG from PLGA NPs in PBS (10 mM, pH 7.4) at 37 °C. Free TMZ and O6BG were used as control. Results are given as mean ± SD (*n* = *3*). An enlargement of the first time points—6 and 24 h—is presented to highlight the burst effect.

For the evaluation of the in vitro release profile of the developed NPs, the dialysis method was used. To simulate the physiological conditions (temperature, salt concentration, and pH), the release studies were conducted at 37 °C in PBS (10 mM, pH 7.4). The release curves were plotted and are shown in Figure 9.

PLGA NPs showed an initial burst release for both drugs, due to the presence of the drug molecules adsorbed at the NPs’ surface. As Figure 9 depicts, at the first 24 h, 48 ± 3% of the total TMZ and 43 ± 3% of the total O6BG were released. Then, both drugs located at the polymeric matrix of the NPs were released in a slower and controlled manner that was prolonged for 20 days.

A faster release for TMZ was verified in comparison to O6BG. The PLGA NPs exhibited a release of TMZ of 81 ± 1% after 20 days, while for O6BG, only about 79 ± 1% of the entrapped drug was released at day 20 (Figure 9). This may occur due to the higher affinity of TMZ to the aqueous buffer (lower log P values). However, the release profile is similar for the two drugs, due to the erosion of the polymer matrix that allows the release of both molecules.

The obtained release profile proved that the developed PLGA NPs are able to maintain the sustained and controlled release of both TMZ and O6BG, and therefore could be used for the co-delivery of entrapped molecules. Further optimization of the release profile to reduce the initial burst release may improve the efficiency of the nanovehicle. 

## 4. Conclusions

PLGA NPs as drug delivery systems have gained increasing interest in the last years, due to their unique physicochemical properties, such as stability, biocompatibility, and biodegradability. Encapsulating drugs in PLGA NPs allows them to maximize their therapeutic effects and to minimize their side effects. Since the biological fate and toxicity of the NPs depend on their physicochemical properties, the developed nanosystems must meet several criteria to be appropriate for brain delivery.

PLGA NPs for the entrapment of both TMZ and O6BG for further GBM treatment were developed in this work. TMZ is the used drug for GBM chemotherapy, and despite its therapeutics effects being well-known, the drug is not able to effectively cure GBM patients. Intrinsic resistance mechanisms are of one of the major obstacles for GBM treatment, due to the DNA repair by the MGMT protein. To overcome this issue, co-therapy with TMZ and an inhibitor of the MGMT protein, O6BG, was proposed. O6BG is a guanine analogue able to inhibit the activity of the MGMT protein, hampering the DNA repair. Thus, O6BG could decrease the resistance to TMZ’s therapy.

Hence, an optimized nanoformulation was developed in this work, and for that, FFD was used. FFD proved to be a suitable approach for the design of co-loaded NPs allowing to optimize the single emulsion solvent evaporation production process with a low replica number in a reliable and precise manner. The regression analysis of the results proved to adequate to identify the main experimental variables affecting the physicochemical properties of the developed NPs. The optimal experimental conditions were chosen, and the optimal nanoformulation was prepared using 1 mg of both drugs, 0.5% (*w*/*v*) of PVA, 1 mL of dichloromethane, and 15 mg of PLGA.

The developed NPs exhibited high encapsulation efficiencies for both drugs and showed a sustained drug release. Thus, it is expected that these nanocarriers will provide an effective brain delivery, allowing the drug to reach the brain at desirable doses, leading to a significant improvement on the GBM treatment.

PLGA NPs provide a novel and potentially efficient approach for the co-administration of TMZ and O6BG, presenting a potential solution for the intrinsic resistance mechanisms to TMZ due to MGMT high expression. Though these NPs could potentially overcome the limitations of the currently available therapies, future in vitro and in vivo tests are necessary to assess the nanosystem efficacy.

## Figures and Tables

**Table 1:** Process and formulation parameters of the used FFD.

Parameter	Component	Units	Applied Level		
			Low Level (−1)	Centre Level (0)	High Level (+1)
**X_1_**	m_TMZ_	mg	0.250	0.625	1
**X_2_**	m_O6BG_	mg	0.250	0.625	1
**X_3_**	%_PVA_	% (*w*/*v*)	2	3	4
**X_4_**	V_DCM_	mL	0.50	0.75	1
**X_5_**	m_PLGA_	mg	10	15	20

Note: m_TMZ_—Mass of TMZ; m_O6BG_—Mass of O6BG; %_PVA_—percent weight per volume of PVA; V_DCM_—Volume of dichloromethane; m_PLGA_—mass of PLGA.

**Table 2:** Outline of the experimental design and results. The experimental levels (low, centre, and high) are represented by the coded values of −1, 0, and +1, respectively.

Run Order	Coded Independent Variables					Measured Dependent Variables				
	X_1_	X_2_	X_3_	X_4_	X_5_	Mean Size (nm)	PdI	Zeta Potential (mV)	EE (%)	
									TMZ	O6BG
**1**	+1	+1	−1	+1	−1	183	0.148	−26.2	55.1	78.5
**2**	−1	+1	+1	−1	−1	202	0.110	−26.7	38.7	74.1
**3**	0	0	0	0	0	173	0.115	−23.4	45.1	71.6
**4**	+1	+1	−1	+1	−1	179	0.165	−24.1	56.7	80.8
**5**	−1	−1	−1	+1	+1	178	0.132	−24.4	53.5	94.0
**6**	−1	+1	−1	−1	+1	193	0.158	−22.4	52.3	91.6
**7**	+1	−1	−1	−1	−1	162	0.158	−27.5	31.8	99.0
**8**	0	0	0	0	0	178	0.127	− 22.1	44.7	77.3
**9**	−1	−1	+1	+1	−1	192	0.190	−27.0	42.6	79.7
**10**	+1	+1	+1	+1	+1	171	0.142	−20.3	27.2	78.5
**11**	+1	−1	+1	−1	+1	200	0.137	−22.7	40.3	99.8
**12**	+1	−1	+1	−1	+1	205	0.109	−22.5	46.3	99.6
**13**	+1	+1	+1	+1	+1	180	0.118	−22.3	28.9	82.6
**14**	+1	−1	−1	−1	−1	176	0.125	−28.0	36.6	99.5
**15**	−1	+1	−1	−1	+1	189	0.130	−21.7	50.9	95.5
**16**	−1	−1	−1	+1	+1	172	0.158	−23.4	54.5	98.0
**17**	−1	−1	+1	+1	−1	184	0.146	−26.2	46.7	78.1
**18**	−1	+1	+1	−1	−1	205	0.141	−27.5	36.5	76.9

**Table 3:** Results of analysis of variance (ANOVA) for the regression models. Non-significant factors are marked as blue.

Source	Size, Y_1_ (nm)				EE of TMZ, Y_2_ (%)				EE of O6BG, Y_3_ (%)			
	SS	df	*F*-Value	*p*-Value	SS	Df	*F*-Value	*p*-Value	SS	df	*F*-Value	*p*-Value
**Model**	4192.98	7	17.06	0.0001	1381.55	7	41.63	<0.0001	4311.36	7	35.96	<0.0001
X_1_	12.20	1	8.92	0.0199	55.29	1	11.66	0.0244	170.83	1	9.97	0.0586
X_2_	117.63	1	14.74	0.0200	41.70	1	8.80	0.0510	190.31	1	11.11	0.0520
X_3_	3452.85	1	30.67	<0.0001	194.24	1	40.97	0.0001	1788.94	1	104.45	<0.0001
X_4_	49.99	1	1.42	0.1263	46.25	1	9.76	0.0208	134.43	1	7.85	0.0745
X_5_	100.42	1	11.41	0.0224	260.82	1	55.02	<0.0001	1843.34	1	107.63	<0.0001
X_2_X_3_	313.15	1	23.14	0.0057	350.60	1	74.20	<0.0001	173.67	1	10.14	0.0577
X_2_X_5_	146.75	1	18.42	0.0176	432.64	1	91.26	<0.0001	9.83	1	0.57	0.8480
**Residual**	351.06	10			47.40	10			171.27	10		
Lack of Fit	46.42	1	1.37	0.2716	2.77	1	0.56	0.4742	29.41	1	1.87	0.2051
Pure Error	304.64	9			44.64	9			141.86	9		
**Cor Total**	4544.04	17			1428.95	17			4482.63	17		
**R^2^**	0,9227				0,9668				0,9618			

**Table 4:** Optimal formulation parameters for the PLGA NPs determined by the experimental design.

Parameter	Component	Units	Optimal Value
**X_1_**	m_TMZ_	mg	1
**X_2_**	m_O6BG_	mg	1
**X_3_**	%_PVA_	% (*w*/*v*)	0.5
**X_4_**	V_DCM_	mL	1
**X_5_**	m_PLGA_	mg	15

**Table 5:** Validation of the model by comparing the predicted values with the observed experimental values. The experimental data is given as the mean ± SD (*n* = 3).

	Size (Y_1_) (nm)	EE TMZ (Y_2_) (%)	EE O6BG (Y_3_) (%)
**Predicted values**	173 (164–180)	68 (63–73)	89 (83–95)
**Experimental values**	177 ± 4	63 ± 4	90 ± 4
**Experimental error**	2%	7%	1%
